# Effects of add-on Celecoxib treatment on patients with schizophrenia spectrum disorders and inflammatory cytokine profile trial (TargetFlame): study design and methodology of a multicentre randomized, placebo-controlled trial

**DOI:** 10.1007/s00702-022-02566-6

**Published:** 2022-11-19

**Authors:** Wolfgang Strube, Aslihan Aksar, Ingrid Bauer, Susana Barbosa, Michael Benros, Christiane Blankenstein, Mattia Campana, Laetitia Davidovic, Nicolas Glaichenhaus, Peter Falkai, Thomas Görlitz, Maximilian Hansbauer, Daniel Heilig, Olfa Khalfallah, Marion Leboyer, Emanuela Martinuzzi, Susanne Mayer, Joanna Moussiopoulou, Irina Papazova, Natasa Perić, Elias Wagner, Thomas Schneider-Axmann, Judit Simon, Alkomiet Hasan

**Affiliations:** 1 Department of Psychiatry, Psychotherapy, and Psychosomatics, Medical Faculty, University of Augsburg, Bezirkskrankenhaus Augsburg, Geschwister-Schönert-Str. 1, 86156 Augsburg, Germany; 2 Institut de Pharmacologie Moléculaire et Cellulaire, Université Côte d’Azur, Centre National de La Recherche Scientifique, Valbonne, France; 3 CORE-Copenhagen Research Centre for Mental Health, Mental Health Centre Copenhagen, Copenhagen University Hospital, Copenhagen, Denmark; 4 Münchner Studienzentrum, School of Medicine, Technical University of Munich, Munich, Germany; 5 Department of Psychiatry and Psychotherapy, University Hospital, LMU Munich, Munich, Germany; 6 Univ Paris Est Créteil, INSERM U955, IMRB, Translational Neuro-Psychiatry Laboratory, AP-HP, Hôpitaux Universitaires Henri Mondor, Département Médico-Universitaire de Psychiatrie Et d’Addictologie (DMU IMPACT), Fédération Hospitalo-Universitaire de Médecine de Précision en Psychiatrie (FHU ADAPT), Fondation FondaMental, 94010 Créteil, France; 7 Department of Health Economics, Center for Public Health, Medical University of Vienna, Vienna, Austria; 8 Department of Psychiatry, University of Oxford, Oxford, UK

**Keywords:** Schizophrenia, Inflammation, Celecoxib, Precision medicine, Targeted therapy, Precision psychiatry

## Abstract

Neuroinflammation has been proposed to impact symptomatology in patients with schizophrenia spectrum disorders. While previous studies have shown equivocal effects of treatments with add-on anti-inflammatory drugs such as Aspirin, *N*-acetylcysteine and Celecoxib, none have used a subset of prospectively recruited patients exhibiting an inflammatory profile. The aim of the study is to evaluate the efficacy and safety as well as the cost-effectiveness of a treatment with 400 mg Celecoxib added to an ongoing antipsychotic treatment in patients with schizophrenia spectrum disorders exhibiting an inflammatory profile. The “Add-on Celecoxib treatment in patients with schizophrenia spectrum disorders and inflammatory cytokine profile trial (TargetFlame)” is a multicentre randomized, placebo-controlled phase III investigator-initiated clinical trial with the following two arms: patients exhibiting an inflammatory profile receiving either add-on Celecoxib 400 mg/day or add-on placebo. A total of 199 patients will be assessed for eligibility by measuring blood levels of three pro-inflammatory cytokines, and 109 patients with an inflammatory profile, i.e. inflamed, will be randomized, treated for 8 weeks and followed-up for additional four months. The primary endpoint will be changes in symptom severity as assessed by total Positive and Negative Syndrome Scale (PANSS) score changes from baseline to week 8. Secondary endpoints include various other measures of psychopathology and safety. Additional health economic analyses will be performed. TargetFlame is the first study aimed at evaluating the efficacy, safety and cost-effectiveness of the antiphlogistic agent Celecoxib in a subset of patients with schizophrenia spectrum disorders exhibiting an inflammatory profile. With TargetFlame, we intended to investigate a novel precision medicine approach towards anti-inflammatory antipsychotic treatment augmentation using drug repurposing.

**Clinical trial registration:** http://www.drks.de/DRKS00029044 and https://trialsearch.who.int/Trial2.aspx?TrialID=DRKS00029044

## Introduction

Despite the significant social and healthcare burden caused by difficult therapy of schizophrenia, no novel antipsychotic concepts have been established in psychiatric care for more than 20 years. In the past 2 years new concepts (such as trace amine-associated receptor 1 (TAAR1) (Koblan et al. 2020) or combination of a muscarinic cholinergic receptor agonist and peripheral antagonist) (Brannan et al. 2021) was introduced as novel antipsychpotic concepts. However, the impact on clinical practice of these early studies is yet unclear. Despite these rather new developments that need to prove efficacy and (cost-)effectiveness in future trials, antipsychotic drugs with established receptor profiles (e.g. antagonism on dopamine D2-receptors or 5-hydroxytryptamine receptors) are the mainstay of treatment for schizophrenia. However, despite their high efficacy for positive symptoms (Huhn et al. 2019) and relapse prevention (Schneider-Thoma et al. 2022), there is a high variability in response across patient populations due to the clinical and biological heterogeneity of the disease. Although national and international guidelines are available to assist clinicians to select and adjust treatment while minimizing the risks of adverse side effects, the process of finding an effective pharmacotherapy is mainly based on individual experience and trial and error procedures. Hence, there is an urgent and yet unmet medical need to identify reliable predictors of treatment response in schizophrenia patients following the concepts of stratified or precision psychiatry (Insel and Cuthbert 2015). Moreover, apart from finding prognostic tools to allocate patients to an optimal treatment scheme, the repurposing of available drugs in terms of establishing new therapeutic concepts on a fast track has been discussed as another possibility to improve treatment of schizophrenia patients. The National Institute of Mental Health (NIMH) defined this multistep process by identifying risk-factors for mental illness based on genetic and biological research, repurposing known compounds in preclinical and animal studies and conducting proof-of-concept trials in patients in academic settings (Brady and Insel 2012; Insel 2012; Hasan et al. 2020). In that regard, targeting neuroinflammation and immune dysregulation to treat schizophrenia is one of those promising pathways (Bishop et al. 2022).

There is ample evidence that immune dysfunction, and more specifically inflammation, is associated in a subgroup with schizophrenia (Muller 2016; Bishop et al. 2022). First, single nucleotide polymorphisms in the human leukocyte antigens (HLA) and complement component 4 (C4) genes are associated with an increased risk of schizophrenia. Second, the prevalence of autoimmune disorders is increased in patients with schizophrenia (Severance et al. 2016). Third, both increased blood cytokine levels and elevated serum levels of C-reactive protein (CRP) have been reported in patients with schizophrenia (Miller et al. 2011). Fourth, in vivo PET imaging (Pasternak et al. 2016) and histopathology (Trepanier et al. 2016) have highlighted neuroinflammation as a hallmark of schizophrenia. Despite accumulating evidence that neuroinflammation contributes to symptoms in schizophrenia, the underlying mechanisms have only begun to be explored. Several studies demonstrate an association between low-grade neuroinflammation and enhanced production of neurotoxic factors, decreased neurotrophic function of microglia and impaired glutamatergic and dopaminergic neurotransmission (de Bartolomeis et al. 2022). Taken together, these data suggest that inflammatory mediators could act directly or indirectly on neural cells inducing or aggravating symptoms. In that regard, treatments with add-on anti-inflammatory drugs have been proposed to improve clinical outcome. This latter hypothesis has been tested in clinical trials in which, e.g. steroids or non-steroid anti-inflammatory drugs, including certain antiphlogistics such as Celecoxib and statins, have been used as add-on treatments (Jeppesen et al. 2020; Cho et al. 2019; Zheng et al. 2017). However, previous findings for psychopathological and other outcomes were heterogenous. Further, none of all previous trials which have been performed so far have selectively targeted patients with an inflammatory profile.

In this context, the effects of add-on Celecoxib treatment in patients with schizophrenia spectrum disorders and inflammatory cytokine profile trial (TargetFlame) is the first clinical study aimed at evaluating the efficacy, safety and cost-effectiveness of the antiphlogistic agent Celecoxib added to an ongoing antipsychotic treatment in a subset of schizophrenia patients exhibiting an inflammatory profile. Inflamed patients will be identified using an unsupervised classification method based on serum levels of the three pro-inflammatory cytokines IL-6, IL-1-beta and TNF-alpha established by a member of our study group. Thus, TargetFlame combines a drug repurposing and a precision medicine approach.

## Methods

### Study design

TargetFlame is a multicentre trial with two German study sites, one French laboratory and one Austrian academic centre, involved. The study is designed as a randomized, double-blind, controlled multicentre phase III clinical trial on a cohort of prospectively recruited patients with a schizophrenia spectrum disorder with add-on health economic analyses. Blood samples will be drawn at the German study sites and serum will be prepared. Every other week, serum samples will be shipped to the French laboratory where they will be immediately assessed for IL-6, TNF-alpha and IL-1-beta: three extendively studied pro-inflammatory cytokines that have been consistently found to be elevated in schizophrenia (Miller et al. 2011). Based on the levels of these cytokines, we will determine the probability that the newly included patient exhibit an inflammatory (“inflamed”) or a non-inflammatory (“non-inflamed”) profile using a cytokine-based algorithm. This algorithm was produced by applying an unsupervised K-mean classification method to a dataset consisting of 289 first-episode-psychosis (FEP) patients of the OPTiMiSE study for which blood levels of IL-6, TNF-alpha and IL-1-beta have been measured (Martinuzzi et al. 2019). Of note, in contrast to many stratification procedures used in medicine, this approach is not based on individual cut-offs. Then inflamed patients will be randomized in two arms and will either receive as investigational medical product (IMP) add-on Celecoxib (daily total dose of 400 mg divided into two daily doses) or add-on placebo. Non-inflamed patients will serve as naturalistic control group without receiving IMP. Regarding the impact of antipsychotics on inflammation, several studies have suggested that antipsychotics may have the following anti-inflammatory properties: First, patients with psychotic disorders exhibited higher levels of proinflammatory cytokines such as IL-6, IL-8, TNFα and IFN-ɣ compared to healthy individuals (Miller et al. 2011; Mondelli et al. 2015); second, levels of these cytokines correlated with symptom severity as well as a poor response to antipsychotics (Mondelli et al. 2015; Enache et al. 2021; Kose et al. 2021); third, antipsychotics reduced the levels of IFN-γ produced by lipopolysaccharide (LPS)-stimulated Peripheral Blood Mononuclear Cells (PBMCs) in vitro (Krause et al. 2013). We anticipate that the patients who will be included in our study will all be treated with antipsychotics. Therefore, it is likely that the levels of proinflammatory cytokines in their blood would have been already lowered by antipsychotics. While this is noteworthy, it does not preclude the use of Celecoxib to further reduce these levels. Thus, the concept and rationale of our study remains valid. The clinical trial has been approved by the local ethics committees and the competent authority in Germany (Federal Institute for Drugs and Medical Device (Bundesinstitut für Arzneimittel und Medizinprodukte (BfArM)). The study was registered in the EU Clinical Trials Register (number: 2021-002572-39), the International Clinical Trials Registry Platform (ICTRP, https://trialsearch.who.int/Trial2.aspx?TrialID=DRKS00029044) and the German DRKS Platform (http://www.drks.de/DRKS00029044) prior to the inclusion of the first patient.

### Study sites

Two clinical sites, one Immunology laboratory and one Department of Health Economy Research, will be involved: the Department of Psychiatry, Psychotherapy, and Psychosomatic Medicine, Faculty of Medicine, University of Augsburg, Bezirkskrankenhaus Augsburg, Germany (coordinating site); the Department of Psychiatry and Psychotherapy, University Hospital, LMU Munich, Germany; and the Institut de Pharmacologie Moléculaire et Cellulaire, Université Côte d’Azur, Valbonne, France. All involved investigators performing patients’ ratings are trained for a standardized evaluation of the patients. Health economics: Department of Health Economics, Center for Public Health, Medical University of Vienna. All involved investigators performing patients’ ratings are trained for a standardized evaluation of the patients.

### Study population

Inclusion criteria are defined as follows: (1) In- and outpatients (men and women) aged between 18 and 65 years with a DSM-V diagnosis of schizophrenia or schizophrenia-spectrum disorder confirmed by the Mini-International Neuropsychiatric Interview (Sheehan et al. 1998). (2) Participants are able to give and sign informed consent, (3) must receive a stable antipsychotic treatment for at least one week, (4) must have a total score ≥ 55 on the Positive and Negative Syndrome Scale (PANSS) (Kay et al. 1987), and (5) must display an "inflamed" serum profile (as defined below). (6) Female participants must have a negative pregnancy test (serum) at baseline and must use a method of contraception that is medically approved by the health authority. (7) Previously unknown rheumatological or hepatitis B virus (HBV)/hepatitis C virus (HCV) disease must be excluded by predefined blood screening tests. The *Rheuma-VOR* app will also be used to exclude potential ommon chronic autoimmune rheumatic diseases (Schwarting et al. 2019). The 1-week criterion regarding stable state of antipsychotics (see 2) was chosen based on the usual time used for antipsychotic-switchting from registrational trials and feasibility approaches allowing to include inpatients prior to hospital discharge.

Exclusion criteria are as follows: (1) incapacity to give informed consent, (2) suicidality or endangerment of others, (3) coercive treatment or forced placement in a psychiatric hospital at the time of study inclusion, (4) medical history of an immune-mediated brain disorder, (5) chronic use of glucocorticosteroids (temporary use is permitted, if stopped at least 1 month before start of treatment trial), (6) chronic use of non-steroidal anti-inflammatory drugs (temporary use is permitted, if stopped at least 1 month before start of treatment trial; on-demand use is permitted only as topic application), (7) current use of statins or other lipid-lowering drugs, (8) current use of tacrolimus or fluconazole, (9) current use of antihypertensives from the substance class of Angiotensin-converting enzyme (ACE)-inhibitors or Angiotensin II-receptor (AT-II) antagonists or beta-blockers with the exception of propranolol, (10) current use of diuretics, (11) current use of carbamazepine, (12) chronic use of antiplatelet agents (e.g. acetylsalicylic acid (ASS), clopidogrel), marcumar or non-vitamin K antagonist oral anticoagulants (NOACs), (13) known history of treatment-resistant hypertension, or known history of ischaemic heart disease, peripheral arterial disease and/or cerebrovascular disease, or known history of congestive heart failure (NYHA class II–IV) or current use of digoxin, (14) known history of type 2 diabetes mellitus, (15) severe liver disorder(s) (serum albumin < 25 g/l or Child–Pugh score > 10), (16) severe kidney disorder(s) (estimated creatinine clearance < 30 ml/min), (17) known history of systemic dermatological disorders, (18) known history of ulcer disease or gastrointestinal bleeding, (19) known history of inflammatory bowel disease, (20) pregnancy or breastfeeding, (21) known history of asthma, acute rhinitis, nasal polyps, angioneurotic edema, urticaria or other allergictype reactions after taking ASS or other non-steroidal anti-inflammatory drugs (NSAIDs) including COX-2 (cyclooxygenase-2)-inhibitors, (22) known hypersensitivity to sulphonamides, known history of hereditary Galactose-intolerance, complete Lactase-deficiency or Glucose-Galactose-malabsorption disorder and (23) intolerance to one of the study drugs. (24) For male participants: pregnancy or breastfeeding of the partner will lead to exclusion. (25) Concurrent enrolment in another clinical trial where the participant is receiving an IMP or participation in another clinical trial with IMP during the last 30 days before inclusion or 7 half-lives of previously used IMP, whichever is longer, is also excluded.

### Intervention

**Fig. 1:**
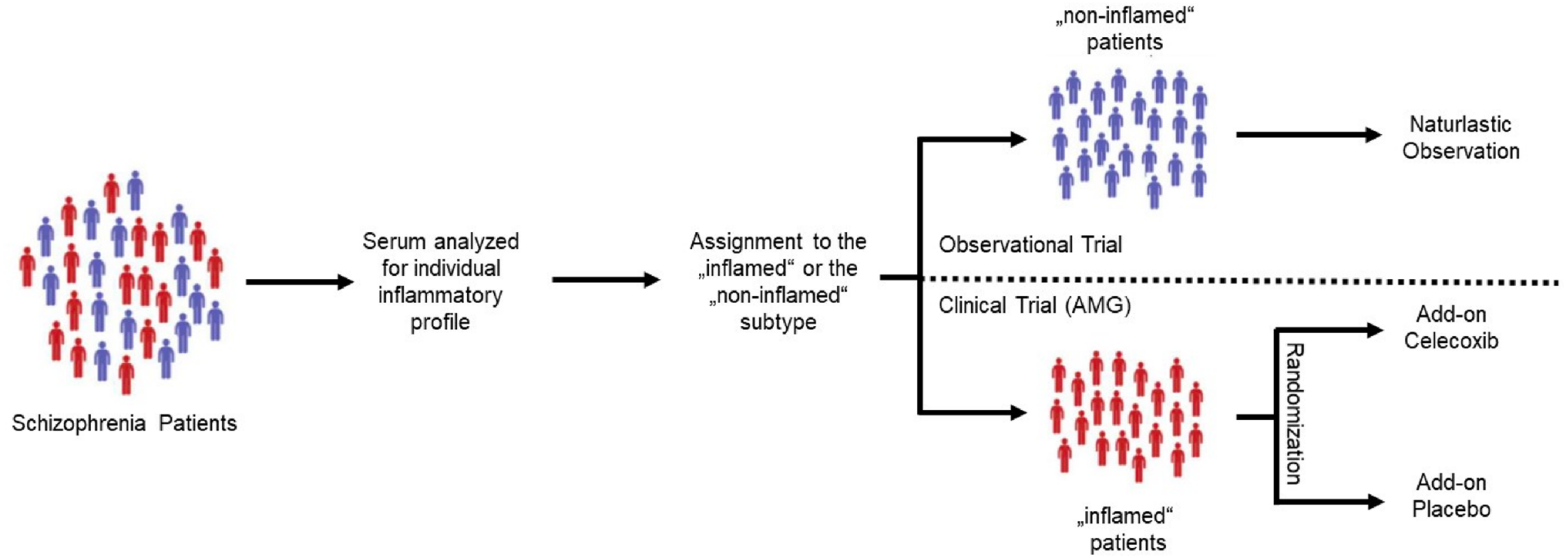
Schematic representation of patient inclusion. Unsupervised classification methods will be used to stratify patients as “inflamed” and “non-inflamed” based on the serum levels of several cytokines. Only patients with an “inflamed profile” will enter the randomized-controlled part of the study and receive either Celecoxib or placebo twice daily

A total of 199 patients will be assessed for eligibility and 109 patients with an inflammatory profile (see power calculation below) will be randomized to either one out of the two interventional groups, add-on Celecoxib 200 mg or placebo twice daily, and eventually treated for 8 weeks. Celecoxib was chosen based on the possibility to be be modified by encapsulation (Pharmacy of the University Hospital Heidelberg), its established safety profile for an 8-week use (Nissen et al. 2016) and the available results from trial on schizophrenia patients without our stratification approach (Jeppesen et al. 2020). Non-inflamed patients will serve as a naturalistic control group (Fig. 1). Participants allocated to the intervention arm will receive two identical capsules per day to maintain blinding. In the interventional group (Celecoxib 400 mg/day), participants will receive 1 capsule with 200 mg Celecoxib in the morning and 1 capsule with 200 mg Celecoxib in the evening. In the control group (placebo), participants will receive two capsules containing placebo each day. Adherence will be assessed by counting the capsules and strategies to improve the adherence include offering contacting patients via phone in cases of no show-up. Patients who will discontinue medication or drop-out for other reasons will be offered to perform the V3 visit (56 days after treatment initiation).

Central randomization will be performed prior to enrolment of the first patient and stratified block wise by study centre at the Münchner-Studien-Zentrum (MSZ). The randomization list will then be forwarded to the pharmacy of the University Hospital Heidelberg (Germany) for labelling and distributing of blinded study medication. Randomization will take place as the patients are assigned the next available patient number in chronological order and receive the corresponding medication kit already containing the correct blinded medication. Safety envelopes for emergency unblinding will be available at both study centres and the integrity of the envelopes will be monitored until study end. To preserve the allocation concealment, the randomization sequence will not be shared with the study personnel (see Table 1 for synopsis of study visits and assessments).

**Table 1:** Study flow-chart

Day	Screening^a^ V0	Baseline^a^ V1	V2	V3	V4	V5
	− 21 to − 1	0–1	14	56	120	180
Intervention of medication		Administration from day 1 to day 56			Follow-up	
Informed consent^b^	X					
Inclusion/exclusion criteria	X	X				
MINI interview	X					
Demographic data/medical/psychiatric history	X					
Pre-study medication	X	X				
Physical examination	X	X	X	X	X	X
Study laboratory^c^	X	X	X	X	X	X
Pregnancy test (screening strip)	X					
Pregnancy test serum		X				
Check of inflammation profile^d,f^	X			X		
Randomization		X				
Interaction medication check	X	X	X	X		
PANSS^e^	X	X		X		X
Andreasen remission criteria		X		X		X
GAF		X		X		X
WHOQoL		X		X		X
Assessment blood pressure and heart rate	X	X	X	X	X	X
Assessment of weight and BMI	X	X	X	X	X	X
ECG^c^	X		X	X		
Study medication (re-) dispensing		X	X			
Study medication return			X	X		
Discontinuation check			X	X	X	X
SAE/AE		X	X	X	X	X
Concomitant medication		X	X	X	X	X
Health economics^f^		X		X		X
Biobanking^f^						

*MINI-Interview* MINI Interview for DSM diagnosis, *PANSS* Positive and Negative Syndrome Scale in Schizophrenia, *GAF* Global Assessment Scale of Functioning, *WHOQoL* WHO-Quality of life assessment, *ECG* electrocardiogram, *BMI* body mass index, *(S)AE* (Serious) Adverse Event

^a^Screening and Baseline can be considered as one visit if both are performed within 7 working days

^b^The ability to give informed consent will be checked again in cases of relevant psychopathological worsening (increase in PANSS total score of ≥25% from baseline)

^c^If ECG or the respective laboratory measures are available from the medical records and were performed within 7 days prior to the respective visit, they can be used for the study

^d^Only patients with an inflamed profile will be randomized. Non-inflamed patients will be invited to participate in V1, V3 and V5

^e^If psychopathological worsening is suspected, additional PANSS ratings should be performed in those visits where PANSS rating is not planned

^f^Participants of this clinical trial are invited to participate in accompanying research by giving separate informed consent

### Endpoints and safety measures

The *primary endpoint* will be change in symptom severity in randomized patients following treatment with add-on Celecoxib compared to control patients following treatment with add-on placebo as assessed by total PANSS score changes from baseline to 8 weeks after treatment initiation. *Secondary endpoints* will include disease severity in the positive, negative, and general psychopathology dimensions assessed using the P-PANSS, N-PANSS and GP-PANSS subscale scores (Kay et al. 1987) after 2 and 6 months compared to baseline. Further, Andreasen Remission criteria (Andreasen et al. 2005) after 2 and 6 months will be compared to baseline. Other measures include level of functioning and quality of life assessed by the Global Assessment of Functioning scale (GAF) score (Endicott et al. 1976) and WHO-Quality of Life assessment (WHOQOL-Group 1998) after 2 and 6 months compared to baseline. Please see Table 1 for more information. Participants of this clinical trial are also invited to participate in accompanying health economic analyses. The cost-effectiveness of the used blood-based algorithm for selecting patients for adjunct anti-inflammatory treatment with Celecoxib will be assessed in a prospective economic evaluation as additional research at the Department of Health Economics, Medical University of Vienna. Costs will be estimated from data on service and other resource uses collected via an adapted German version of the HEQ (Kingslake et al. 2017; Simon and Mayer 2016), at baseline, 2-month follow-up and 6-month follow-up. Outcomes will be assessed in terms of Quality-Adjusted Life Years (QALYs) (Drummond et al. 2005) calculated using a the EuroQoL EQ-5D-5L health-related quality of life instrument (EuroQol 1990) based on the German value set (Ludwig et al. 2018), and in terms of broader wellbeing expressed as Capability-Weighted Life Years (CWLYs) (Simon et al. 2021) based on the Oxford CAPabilities questionnaire-Mental Health (OxCAP-MH) instrument (German version) (Simon et al. 2018, 2013; Laszewska et al. 2019). Finally, patients can opt to participate in accompanying biobanking, where serum samples will be collected and stored for future biological analyses at the LMU Department of Psychiatry and Psychotherapy Biobank.

*Safety measures* include physical examinations, electrocardiography (ECG), study laboratory, blood pressure and heart frequency, as well as the assessment of height, body weight and body mass index (BMI). Due to the specific side-effect profile of Celecoxib, special attention is paid regarding the assessment of standard blood count, creatinine, liver enzymes (ASAT, ALAT), serum albumin, potassium and sodium, which are assessed at each study visit at baseline, during the intervention period, and during follow-up (see Table 1). Adverse events (AE), severe adverse events (SAE) and suspected unexpected serious adverse reactions (SUSAR) are documented following established definitions and legal requirements. The intensity of AEs is defined according to the common terminology criteria for adverse events (CTCAE Version 4.0, https://www.eortc.be/services/doc/ctc/CTCAE_4.03_2010-06-14_QuickReference_5x7.pdf) (Savarese 2013). During the follow-up period (starts after V3), hospitalization to a psychiatric hospital due to the patient`s psychiatric illness was a priori defined, not to be considered as a SAE.

### Sample size justification and planned data analysis strategy

Assuming as analysis method a RM-ANOVA with the within-subject variable time (before and after intervention [baseline, week 8]) and the between-group variable group (“add-on Celecoxib” versus “add-on placebo”) with *f* = 0.285 and a significance level of *α* = 0.05, a power of 1—*β* = 0.8 and a correlation between the measurements of *r* = 0.5, we calculated that we would need 38 patients (completers) per group (76 in total) to detect differences between groups (G*Power 3.1.9 was used for sample size calculation (Faul et al. 2007)). As we will perform an ITT analysis (linear mixed model analysis, if normality and variance homogeneity assumptions will be fulfilled), 76 patients is the aspired count of patients with 8-week primary endpoint data. Assuming a drop-out rate of 30%, 109 patients (76/0.7 ≈ 109) will have to be randomized. Based on preliminary data showing that 55% of patients with schizophrenia exhibit an inflammatory profile (Glaichenhaus’ laboratory), we have calculated that (109/0.55 ≈ 199) should be screened. If the distribution of inflamed/non-inflamed participants does not follow this assumption, more patients will need to be screened to reach the inclusion of 109 patients with an inflamed profile. The intention-to-treat (ITT) population includes all patients with an inflamed profile who received at least one dose of IMP. The per protocol (PP) population will include all participants evaluable for the primary endpoint without major protocol violations. The safety population (SP) consists of all patients who entered the trial and will be used for conducting all safety analyses (in accordance with the ITT population).

A statistical analysis plan will be developed before any analyses will be performed. For the primary endpoint analysis, a linear mixed model non-restrictively assuming an unstructured covariance matrix will be performed with fixed factors group, time and ‘group x time’ interactions. Analyses will be adjusted for centre, BMI, age and gender as obesity, age and gender may impact cytokine levels. Moreoever, an adjustment of those analyses for PANSS total baseline scores in the case of significant group differences will be performed. Baseline differences will be analyses with independent *t* test or Mann–Whitney *U* test. Continuous secondary endpoints will be analysed using linear mixed models adjusted for centre, age, BMI, gender and, if necessary, for related baseline values and with subsequent parametric post-hoc tests if the requirements for this strategy are met (tested by Kolmogorov–Smirnov tests and Levene’s tests). In cases where the assumptions of normality or variance homogeneity are violated, a monotonic continuous variable transformation will be performed. If this first step is not successful, corresponding non-parametric tests will be used. Additionally, binary logistic regression analysis including a factor variable for centres will be performed. Side-effects, AE and SAE will be analysed with descriptive statistics and likelihood-ratio tests. Demographic information will be shown for each group separately. Dichotomous variables will be analysed with likelihood-ratio tests and continuous variables will be analysed using analyses of variance or respective non-parametric tests. All tests of secondary endpoints will be computed in the ITT and per protocol (PP) populations in an explorative manner on a two-sided significance level of 5%. Outcome data of inflamed patients who participated in the double-blind treatment phase will be compared to the outcome data of the non-inflamed patients (naturalistic control group) in an exploratory manner. An interim analysis is not planned.

The primary economic evaluation (add-on study) will be in the form of a within-trial incremental cost–utility analysis appended with further cost-effectiveness analyses conducted both from a health and social care perspective and from a societal perspective (Drummond et al. 2005). All health economic analyses will be based on the ITT sample with multiple imputation of missing data together with extensive sensitivity and uncertainty analyses.

### Organizational framework

Organizational project management, safety management, monitoring and data management is performed by the Münchner Studienzentrum (MSZ), an academic clinical research organization of the Technical University of Munich, School of Medicine. A detailed description of this framework is available elsewhere (Hasan et al. 2020). For safety monitoring, an independent safety monitoring board (SMB) with a SMB Charta has been established. The documentation of the study data in adherence to the GCP-guidelines and the clinical trial protocol is the responsibility of the investigator and will be performed in a certified clinical trial database (Macro 6). Original data (source documents) remain in hospital medical records and information on the case report form must be traceable and consistent with the original data. Original written informed consent signed by the patient is kept by the investigator and a signed copy will be given to the patient. All study procedures are in accordance with the guidelines of Good Clinical Practice (GCP) of the international conference on harmonization of technical requirements for registration of pharmaceuticals for human use (ICH), and the principles of the Declaration of Helsinki. All sites agreed to adhere to the instructions and procedures described in the study protocol and thereby to adhere to the principles of ICH-GCP. The most recent version of the study protocol is available at http://www.drks.de/DRKS00029044.

At the time of this article submission, the clinical trial is open for recruitment, which began late July 2022. Until now, six participants have been enrolled.

## Discussion

TargetFlame combines a precision psychiatry and a drug repurposing approach in which patients with an inflammatory phenotype are selected and treated with an immunomodulatory drug. The immune hypothesis of schizophrenia (Muller 2016; Bishop et al. 2022) poses one promising alternative to the classic dopamine hypothesis of schizophrenia. This concept allows to investigate therapeutic approaches beyond established pathways targeted by antipsychotics. Personalized medicine is already becoming a reality in cancer therapy where molecular diagnosis is leading more and more to tailored treatments with improved outcomes. In contrast to other medical specialties, diagnosis and prognosis in psychiatry are despite interesting approaches [e.g. multimodal machine learning outcome prediction (Koutsouleris et al. 2021)] still limited to the assessment of subjective and observable symptoms. Regarding concepts using multimodal data-integration, our approach in schizophrenia is based on a simple blood test, which could be, if successful, performed by many laboratories. Our project builds upon solid data indicating that immune-related biomarkers, and especially markers of inflammation, could be used as predictors for treatment response in psychosis patients (Martinuzzi et al. 2019) and that schizophrenia patients could readily be stratified into two clinically distinct clusters based on serum levels of several pro-inflammatory cytokines. A meta-analysis performed by members of our group showed that the general concept of repurposing anti-inflammatory drugs in addition to an antipsychotic treatment is of interest. This meta-analysis identified a total of 70 randomized controlled trials with 4104 participants and showed that adding an anti-inflammatory agent to antipsychotic treatment was superior to adding placebo (SMD = − 0.29; 95% CI = − 0.40 to − 0.19). However, the results for the established NSAIDs that have the potential to be repurposed for a broad use in clinical practice due to our long-standing experience with those compounds showed heterogenous results (Jeppesen et al. 2020). An analysis of the efficacy of repurposing NSAIDs (ASS, Celecoxib) based on five studies and 138 (active compound) vs. 124 (placebo) patients showed a relevant between-study heterogeneity and no overall effect (95% CI = − 11.43 to 3.25) (Jeppesen et al. 2020). Thus, there is an urgent need for tailored treatment approaches focusing on those patients who show relevant peripheral inflammation rather than adding a NSAID to every patient with schizophrenia with ongoing symptoms.

Using Celecoxib for the proposed indication demands a detailed risk–benefit evaluation. Celecoxib has been approved and is indicated according to the *Summary of product characteristics* (SmPC) for treatment of osteoarthritis, rheumatoid arthritis, juvenile rheumatoid arthritis in patients 2 years and older, ankylosing spondylitis, acute pain and primary dysmenorrhea. TargetFlame will repurpose Celecoxib for the treatment of schizophrenia. Patients will be treated with their usual antipsychotics with additional health monitoring through multiple clinical examinations as well as blood tests and electrocardiogram (ECG) that go beyond the safety assessment in clinical practice. Using an IMP such as Celecoxib may be associated with drug-specific side effects, which will be assessed through collection of safety parameters. Of note, Celecoxib has a favourable side effect profile with serious adverse reactions being very rare according to the Summary of Product Characteristics (< 1/10,000). Additionally, and in contrast to newly developed and untested molecules, Celecoxib has been used for many years in other indications and long-term dosage of 400 mg per day, thus offering the advantage of having been extensively tested in large study populations. Since Celecoxib is almost exclusively metabolized through the liver and excreted in the form of inactive metabolites via the faeces and the kidneys, tests of liver and kidney function will be performed. Broad exclusion criteria focussing on the main risks associated with Celecoxib such as cardiovascular event, gastrointestinal or hematologic side-effects have been implemented. The cardiovascular safety of Celecoxib was shown to be not inferior to other NSAIDs (Nissen et al. 2016). Moreover, most risks are associated with a long-term use of Celecoxib, and we will apply the IMP for a maximum of eight weeks. Our precision psychiatry approach will further reduce the individual risk as only those patients who have an inflammatory profile will be included. Until now, studies using anti-inflammatory drugs to treat schizophrenia (e.g. Celecoxib, ASS, *N*-acetylcysteine, the antibiotic minocycline, the neurosteroid pregnenolone or Dehydroepiandrosterone (DHEA), or monoclonal antibodies such as tocilizumab) [for review see (Jeppesen et al. 2020)] did not stratify according to an individual phenotype. Such approach is also expected to improve cost-effectiveness.

## Conclusions

The goal of the Target Flame trial was to evaluate the effects of an add-on treatment with the anti-inflammatory drug Celecoxib in a subset of schizophrenia patients exhibiting an inflammatory profile. To this aim, we will conduct an exploratory randomized, double-blind phase III clinical trial in prospectively recruited patients with schizophrenia spectrum disorders. Inflamed patients will be randomized in two arms and treated with either add-on Celecoxib or add-on placebo. Clinical assessments and health economic assessments will be performed at baseline and after 2 months (end of intervention) and 6 months (follow-up). TargetFlame is the first trial in schizophrenia research combining stratified treatment and drug repurposing approaches.

## Data Availability

Data sharing not applicable to this article as no datasets were generated or analysed during the current study.

## References

[CR1] Andreasen NC, Carpenter WT, Kane JM, Lasser RA, Marder SR, Weinberger DR (2005). Remission in schizophrenia: proposed criteria and rationale for consensus. Am J Psychiatry.

[CR2] Bishop JR, Zhang L, Lizano P (2022). Inflammation subtypes and translating inflammation-related genetic findings in schizophrenia and related psychoses: a perspective on pathways for treatment stratification and novel therapies. Harv Rev Psychiatry.

[CR3] Brady LS, Insel TR (2012). Translating discoveries into medicine: psychiatric drug development in 2011. Neuropsychopharmacology.

[CR4] Brannan SK, Sawchak S, Miller AC, Lieberman JA, Paul SM, Breier A (2021). Muscarinic cholinergic receptor agonist and peripheral antagonist for schizophrenia. N Engl J Med.

[CR5] Cho M, Lee TY, Kwak YB, Yoon YB, Kim M, Kwon JS (2019). Adjunctive use of anti-inflammatory drugs for schizophrenia: a meta-analytic investigation of randomized controlled trials. Aust N Z J Psychiatry.

[CR6] de Bartolomeis A, Barone A, Vellucci L, Mazza B, Austin MC, Iasevoli F, Ciccarelli MJMN (2022) Linking inflammation, aberrant glutamate-dopamine interaction, and post-synaptic changes: translational relevance for schizophrenia and antipsychotic treatment: a systematic review. Mol Neurobiol Mol Neurobiol. 2022;59(10):6460–6501. 10.1007/s12035-022-02976-3 PMC9463235 35963926

[CR7] Drummond ME, Sculpher MJ, Torrance GW (2005). Methods for the economic evaluation of health care programmes.

[CR8] Enache D, Nikkheslat N, Fathalla D, Morgan BP, Lewis S, Drake R, Deakin B, Walters J, Lawrie SM, Egerton A, MacCabe JH, Mondelli V (2021). Peripheral immune markers and antipsychotic non-response in psychosis. Schizophr Res.

[CR9] Endicott J, Spitzer RL, Fleiss JL, Cohen J (1976). The global assessment scale. A procedure for measuring overall severity of psychiatric disturbance. Arch Gen Psychiatry.

[CR10] EuroQol G (1990). EuroQol—a new facility for the measurement of health-related quality of life. Health Policy.

[CR11] Faul F, Erdfelder E, Lang AG, Buchner A (2007). G*Power 3: a flexible statistical power analysis program for the social, behavioral, and biomedical sciences. Behav Res Methods.

[CR12] Hasan A, Roeh A, Leucht S, Langguth B, Hansbauer M, Oviedo-Salcedo T, Kirchner SK, Papazova I, Lohrs L, Wagner E, Maurus I, Strube W, Rossner MJ, Wehr MC, Bauer I, Heres S, Leucht C, Kreuzer PM, Zimmermann S, Schneider-Axmann T, Gorlitz T, Karch S, Egert-Schwender S, Schossow B, Rothe P, Falkai P (2020). Add-on spironolactone as antagonist of the NRG1-ERBB4 signaling pathway for the treatment of schizophrenia: Study design and methodology of a multicenter randomized, placebo-controlled trial. Contemp Clin Trials Commun.

[CR13] Huhn M, Nikolakopoulou A, Schneider-Thoma J, Krause M, Samara M, Peter N, Arndt T, Backers L, Rothe P, Cipriani A, Davis J, Salanti G, Leucht S (2019). Comparative efficacy and tolerability of 32 oral antipsychotics for the acute treatment of adults with multi-episode schizophrenia: a systematic review and network meta-analysis. Lancet.

[CR14] Insel TR (2012). Next-generation treatments for mental disorders. Sci Transl Med.

[CR15] Insel TR, Cuthbert BN (2015). Medicine. Brain disorders? Precisely. Science.

[CR16] Jeppesen R, Christensen RHB, Pedersen EMJ, Nordentoft M, Hjorthoj C, Kohler-Forsberg O, Benros ME (2020). Efficacy and safety of anti-inflammatory agents in treatment of psychotic disorders—a comprehensive systematic review and meta-analysis. Brain Behav Immun.

[CR17] Kay SR, Fiszbein A, Opler LA (1987). The positive and negative syndrome scale (PANSS) for schizophrenia. Schizophr Bull.

[CR18] Kingslake J, Dias R, Dawson GR, Simon J, Goodwin GM, Harmer CJ, Morriss R, Brown S, Guo B, Dourish CT, Ruhe HG, Lever AG, Veltman DJ, van Schaik A, Deckert J, Reif A, Stablein M, Menke A, Gorwood P, Voegeli G, Perez V, Browning M (2017). The effects of using the PReDicT Test to guide the antidepressant treatment of depressed patients: study protocol for a randomised controlled trial. Trials.

[CR19] Koblan KS, Kent J, Hopkins SC, Krystal JH, Cheng H, Goldman R, Loebel A (2020). A non-D2-receptor-binding drug for the treatment of Schizophrenia. N Engl J Med.

[CR20] Kose M, Pariante CM, Dazzan P, Mondelli V (2021). The role of peripheral inflammation in clinical outcome and brain imaging abnormalities in psychosis: a systematic review. Front Psychiatry.

[CR21] Koutsouleris N, Dwyer DB, Degenhardt F, Maj C, Urquijo-Castro MF, Sanfelici R, Popovic D, Oeztuerk O, Haas SS, Weiske J, Ruef A, Kambeitz-Ilankovic L, Antonucci LA, Neufang S, Schmidt-Kraepelin C, Ruhrmann S, Penzel N, Kambeitz J, Haidl TK, Rosen M, Chisholm K, Riecher-Rossler A, Egloff L, Schmidt A, Andreou C, Hietala J, Schirmer T, Romer G, Walger P, Franscini M, Traber-Walker N, Schimmelmann BG, Fluckiger R, Michel C, Rossler W, Borisov O, Krawitz PM, Heekeren K, Buechler R, Pantelis C, Falkai P, Salokangas RKR, Lencer R, Bertolino A, Borgwardt S, Noethen M, Brambilla P, Wood SJ, Upthegrove R, Schultze-Lutter F, Theodoridou A, Meisenzahl E, Consortium P (2021). Multimodal machine learning workflows for prediction of psychosis in patients with clinical high-risk syndromes and recent-onset depression. JAMA Psychiat.

[CR22] Krause D, Weidinger E, Dippel C, Riedel M, Schwarz MJ, Muller N, Myint AM (2013). Impact of different antipsychotics on cytokines and tryptophan metabolites in stimulated cultures from patients with schizophrenia. Psychiatr Danub.

[CR23] Laszewska A, Schwab M, Leutner E, Oberrauter M, Spiel G, Simon J (2019). Measuring broader wellbeing in mental health services: validity of the German language OxCAP-MH capability instrument. Qual Life Res.

[CR24] Ludwig K, von der Schulenburg J-MG, Greiner W (2018). German value set for the EQ-5D-5L. Pharmacoeconomics.

[CR25] Martinuzzi E, Barbosa S, Daoudlarian D, Bel Haj Ali W, Gilet C, Fillatre L, Khalfallah O, Troudet R, Jamain S, Fond G, Sommer I, Leucht S, Dazzan P, McGuire P, Arango C, Diaz-Caneja CM, Fleischhacker W, Rujescu D, Glenthoj B, Winter I, Kahn RS, Yolken R, Lewis S, Drake R, Davidovic L, Leboyer M, Glaichenhaus N, Group OPS (2019). Stratification and prediction of remission in first-episode psychosis patients: the OPTiMiSE cohort study. Transl Psychiatry.

[CR26] Miller BJ, Buckley P, Seabolt W, Mellor A, Kirkpatrick B (2011). Meta-analysis of cytokine alterations in schizophrenia: clinical status and antipsychotic effects. Biol Psychiatry.

[CR27] Mondelli V, Ciufolini S, BelvederiMurri M, Bonaccorso S, Di Forti M, Giordano A, Marques TR, Zunszain PA, Morgan C, Murray RM, Pariante CM, Dazzan P (2015). Cortisol and inflammatory biomarkers predict poor treatment response in first episode psychosis. Schizophr Bull.

[CR28] Muller N (2016). What role does inflammation play in schizophrenia?. Expert Rev Neurother.

[CR29] Nissen SE, Yeomans ND, Solomon DH, Luscher TF, Libby P, Husni ME, Graham DY, Borer JS, Wisniewski LM, Wolski KE, Wang Q, Menon V, Ruschitzka F, Gaffney M, Beckerman B, Berger MF, Bao W, Lincoff AM, Investigators PT (2016). Cardiovascular safety of celecoxib, naproxen, or ibuprofen for arthritis. N Engl J Med.

[CR30] Pasternak O, Kubicki M, Shenton ME (2016). In vivo imaging of neuroinflammation in schizophrenia. Schizophr Res.

[CR31] Savarese DJUW (2013) Common terminology criteria for adverse events. UpToDate, Waltham, pp 1–9

[CR32] Schneider-Thoma J, Chalkou K, Dorries C, Bighelli I, Ceraso A, Huhn M, Siafis S, Davis JM, Cipriani A, Furukawa TA, Salanti G, Leucht S (2022). Comparative efficacy and tolerability of 32 oral and long-acting injectable antipsychotics for the maintenance treatment of adults with schizophrenia: a systematic review and network meta-analysis. Lancet.

[CR33] Schwarting A, Dreher M, Assmann G, Witte T, Hoeper K, Schmidt RE (2019). Experiences and results from Rheuma-VOR. Z Rheumatol.

[CR34] Severance EG, Yolken RH, Eaton WW (2016). Autoimmune diseases, gastrointestinal disorders and the microbiome in schizophrenia: more than a gut feeling. Schizophr Res.

[CR35] Sheehan DV, Lecrubier Y, Sheehan KH, Amorim P, Janavs J, Weiller E, Hergueta T, Baker R, Dunbar GC (1998) The Mini-International Neuropsychiatric Interview (M.I.N.I.): the development and validation of a structured diagnostic psychiatric interview for DSM-IV and ICD-10. J Clin Psychiatry 59 Suppl 20:22–33 (quiz 34–57) 9881538

[CR38] Simon J, Mayer S (2016) HEQ (Health Economics Questionnaire), Version 08-09-2016. Department of Health Economics, Center for Public Health, Medical University of Vienna, Austria. 10.5281/zenodo.4559790

[CR36] Simon J, Anand P, Gray A, Rugkasa J, Yeeles K, Burns T (2013). Operationalising the capability approach for outcome measurement in mental health research. Soc Sci Med.

[CR37] Simon J, Laszewska A, Leutner E, Spiel G, Churchman D, Mayer S (2018). Cultural and linguistic transferability of the multi-dimensional OxCAP-MH capability instrument for outcome measurement in mental health: the German language version. BMC Psychiatry.

[CR39] Simon J, Mayer S, Laszewska A, Rugkasa J, Yeeles K, Burns T, Gray A (2021). Cost and quality-of-life impacts of community treatment orders (CTOs) for patients with psychosis: economic evaluation of the OCTET trial. Soc Psychiatry Psychiatr Epidemiol.

[CR40] Trepanier MO, Hopperton KE, Mizrahi R, Mechawar N, Bazinet RP (2016). Postmortem evidence of cerebral inflammation in schizophrenia: a systematic review. Mol Psychiatry.

[CR41] WHOQOL-Group (1998). Development of the World Health Organization WHOQOL-BREF quality of life assessment. Psychol Med.

[CR42] Zheng W, Cai DB, Yang XH, Ungvari GS, Ng CH, Muller N, Ning YP, Xiang YT (2017). Adjunctive celecoxib for schizophrenia: a meta-analysis of randomized, double-blind, placebo-controlled trials. J Psychiatr Res.

